# Awareness of Age-Related Change as a Behavioral Determinant of Survival Time in Very Old Age

**DOI:** 10.3389/fpsyg.2021.727560

**Published:** 2021-09-28

**Authors:** Roman Kaspar, Hans-Werner Wahl, Manfred Diehl

**Affiliations:** ^1^Cologne Center for Ethics, Rights, Economics, and Social Sciences of Health, University of Cologne, Cologne, Germany; ^2^Network Aging Research of Heidelberg University, Heidelberg, Germany; ^3^Department of Human Development and Family Studies, Colorado State University, Fort Collins, CO, United States

**Keywords:** advanced old age, views on aging, cognitive impairment, valuation of life, agency, control beliefs, obsolescence, survival

## Abstract

Although research on the association between subjective views of aging (VOA) and survival is scarce, more negative VOA have been found to be associated with increased all-cause mortality, even after controlling for possible confounders. Longitudinal studies on the predictive association of VOA with survival in individuals aged 80 years or older are, however, very limited. Thus, the aim of this study was to link adults’ awareness of age-related change (AARC), a multidimensional measure of adults’ subjective VOA, to survival time across a 3.5-year observation interval in advanced old age. To put the AARC construct in context, the study also considered related psychosocial concepts (i.e., perceived control and appraisal of life) essential for coping with late-life challenges as potential behavioral predictors of longevity. Data came from a representative panel study that included persons living in community and institutional settings. A total of 1,863 interviews were conducted at wave 1. This study used meta-data from wave 2 fieldwork 2 years after the initial assessment and death records obtained during panel maintenance after 3.5 years to estimate determinants of survival. Results showed that loss-related VOA indicated increased risk to survival, whereas gain-related VOA were predictive of longer survival. Both perceived age-related losses and perceived age-related gains exerted a significant independent effect on late-life mortality over and above socio-demographic background characteristics, perceived control, engagement with life, as well as health status. These findings suggest that the multidimensional examination of very old adults’ VOA may help to better understand successful longevity in the Fourth Age.

## Introduction

Advanced old age is frequently seen as the most vulnerable period of the human lifespan because many adults experience multimorbidity, functional disability, motor and sensory impairment, significant cognitive decline, and frailty. Psychological conceptualizations of what has also been named the “Fourth Age” to a large extent echo this biomedical loss perspective of very late life, resulting in what Baltes and Smith (2003) have described as the “dilemmas of the fourth age” to be contrasted with the successes of the Third Age (Baltes and Smith, 2003; Wahl and Ehni, 2020). Still, psychological resources to counteract and cope with increasing health-related and functional losses seem not to be completely exhausted in the Fourth Age. For example, the phenomenon of the subjective age bias (i.e., individuals feeling significantly younger than their chronological age) persists into advanced old age and the gap between felt age and chronological age, in fact, increases considerably in very old age (Pinquart and Wahl, 2021). In addition, subjective health evaluations which are of utmost importance for quality of life at large depict relatively little or even no decline, although objective health tends to decline in pronounced ways (Wettstein et al., 2016). Both findings have been shown to be quite robust and suggest that very old adults manage to distance themselves, at least to some extent, quite effectively from the down sides of the Fourth Age.

This article addresses whether psychological resources also account for significant variance in predicting survival in advanced old age. An important background for addressing this question is that the predictive strength of social factors, such as income, education, and marital status, for survival is much weaker in those 80+ than in younger age groups (Goldman et al., 1995; Dupre, 2007). In addition, a recent analysis based on large samples from the National Health and Nutrition Examination Survey (NHANES) data infrastructure revealed that although instrumental activities of daily living (IADL) and self-rated health were the most important predictors of survival in those in the Third (65–79 years) and the Fourth Age (80 years and older), their incremental predictive value decreased considerably from the Third to the Fourth Age group (Goldman et al., 2017). Finally, there is a general scarcity of studies identifying psychological predictors of survival in very old age. That is, data are missing whether the “age-as leveler” hypothesis (Goldman et al., 2017) not only applies to social and health-related factors, but also to psychological resources/risk factors. In other words, is the high likelihood of biological frailty in advanced old age canceling out the effects of psychological factors that are important predictors of survival in earlier stages of old age?

Against this background, the primary aim of this study was to link awareness of age-related change (AARC), an established concept to assess older adults’ subjective views of aging (VOA) in a multidimensional way (Diehl et al., 2021), to survival time across a 3.5-year interval in a large sample of individuals aged 80 years and older. To put the AARC concept in perspective, the study also considered related psychosocial resources essential for coping with the challenges of the Fourth Age, such as retaining a positive appraisal of life (e.g., purpose in life and optimism) and a sense of control over one’s life (e.g., mastery and perceived restrictions), as potential behavioral determinants of longevity. These psychosocial resources complemented established survival predictors (e.g., age, gender, ADL, and subjective health) to provide an overall more comprehensive picture of predicting survival in very late life.

Considering in our study the connection between subjective VOA and late-life survival is based on the observation that individuals reflect on their own development and try to understand their own aging as they move across the adult lifespan (Brandtstädter and Rothermund, 2002; Diehl et al., 2015; Kornadt et al., 2019). Thus, aside from using chronological age as a marker of their position in the life course (Settersten and Hagestad, 2015), individuals also draw on their perceptions, and behavioral experiences (Miche et al., 2014b) to establish VOA as part of their identity (Diehl et al., 2021).

A vast body of research has documented that more negative VOA are associated with a range of unfavorable developmental outcomes, such as poorer physical and mental health, and poorer cognitive functioning, including cognitive pathology (for review, see Westerhof et al., 2014; Diehl et al., 2015, 2021; Wurm et al., 2017). A central aspect for this article is that more negative VOA have also been found to be associated with increased all-cause mortality, even after controlling for confounding variables (Kotter-Grühn et al., 2009; Westerhof et al., 2014; Stephan et al., 2018). Yet, to the best of our knowledge, only two earlier publications have addressed the association between VOA in very old age and survival and both were based on data from the same study (i.e., Berlin Aging Study). Maier and Smith (1999) found in a sample of individuals 70 years and older at baseline that lowered dissatisfaction with one’s own aging remained a significant predictor of survival time based on survival status data gathered 3 to 6 years after baseline assessment. Importantly, no effect of chronological age was observed; that is, dissatisfaction with aging maintained its role as a significant predictor also in those 85 years and older. Kotter-Grühn et al. (2009) showed that dissatisfaction with one’s own age was more strongly related to time-to-death, whereas, the degree of feeling younger than one’s own age was more closely associated with chronological age (i.e., distance from birth). In summary, longitudinal findings on VOA in very old age are very limited. Furthermore, given the importance of multidimensional assessment of VOA (Diehl et al., 2021) reflecting losses but also gains, it seems critical to examine multiple, distinct dimensions of VOA. Finally, to better understand the relevance of VOA for survival in advanced old age, it is also important to consider them in combination with other essential indicators of psychosocial functioning with significance for survival.

Views of aging have been assessed in multiple ways in the literature, including felt age, attitudes toward own aging, or aging satisfaction (for an overview, see Diehl et al., 2014; Klusmann et al., 2020). This study relied on a relatively recent and multi-dimensional conceptualization and measurement of VOA. Specifically, Diehl and Wahl’s (2010) construct of awareness of age-related change (AARC) was used in the present study. Diehl and Wahl (2010) defined AARC as “all those experiences that make a person aware that his or her behavior, level of performance, or ways of experiencing his or her life have changed as a consequence of having grown older (i.e., increased chronological age)” (p. 340). We assumed that such an experience-based reflection on one’s own aging should be particularly pronounced in very old age, as this is a period of life when severe age-related losses may become normative for many individuals (Baltes and Smith, 2003; Wahl and Ehni, 2020). We also expected that the two major dimensions of AARC (i.e., perceived age-related gains and perceived age-related losses) would be a particularly well-suited construct in the VOA domain to predict survival in very old adults.

First, Diehl and Wahl’s (2010) conceptualization of AARC and its measurement relies on *actual perceptions* of changed behavior, changed performance, or changed personal experiences. Thus, in contrast to other measurement approaches (e.g., the attitudes toward own aging approach, ATOA; see Diehl et al., 2014; Miche et al., 2014a) the AARC questionnaire avoids general ratings of a person’s perceptions of aging and asks for ratings regarding specific behaviors and experiences in critical life domains. Given the many day-to-day changes coming with very old age and accumulating challenges in gait, sensory, motor functioning, and out-of-home behavior (Baltes and Smith, 2003), the AARC questionnaire seemed particularly suited to assess VOA in very old adults. At the same time, given its focus on actual perceptions of day-to-day behaviors, AARC may be particularly sensitive to capture self-perceived changes in functions and performances that may be indicative of serious declines and may signal impending death.

Second, the AARC conceptualization was from its inception designed as a multidimensional approach, differentiating between both positive (AARC-Gains) and negative (AARC-Losses) perceptions and interpretations of events, behaviors and sensations across various life domains (e.g., health, social relationships, leisure, and lifestyle). Previous research in younger age groups has shown that perceived age-related gains and losses co-exist even within behavioral domains and have different antecedents and different associations with developmental outcomes, including depression, psychological well-being, and self-rated health (Miche et al., 2014b; Brothers et al., 2016, 2019, 2021; Dutt et al., 2018a,b; Kaspar et al., 2019; Diehl et al., 2021). Thus, AARC explicitly addresses gains as a developmental option in very old age (Baltes and Smith, 2003; Baltes et al., 2006). More concretely, the concept of AARC captures, on the one hand, the “success” of having survived many peers, but on the other hand also the fact that many individuals have to cope with serious declines and potential impairments in biological and functional capacity. In very old age, however, age-related losses may become more pronounced and it may become increasingly harder to appreciate the benefits that aging brought about. In fact, the strength and vulnerability integration model (SAVI, Charles, 2010) proposes that age-related gains might no longer be sufficient to retain optimal functioning and well-being in the Fourth Age. With respect to the prediction of survival, however, perceptions of age-related gains may be particularly important, because they may reflect motivational resources that may be activated in drawing on remaining reserve capacities and sources of resilience.

Important for assessing AARC in very old individuals, Kaspar et al. (2019) developed a short form of the AARC questionnaire specifically for use in large-scale surveys and in populations in which the administration of lengthy questionnaires is not feasible. This short form was used in the present study. Like for the long form (Brothers et al., 2019), the two-factor solution was confirmed using confirmatory factor analysis and independent samples (Kaspar et al., 2019; Sabatini et al., 2020). Findings for the AARC measure suggest that VOA in very old age are both a result of change in health status and engagement in life and a predictor of future health status (Spuling et al., 2013; Dutt et al., 2018a; Kaspar et al., 2021). However, no studies are currently available addressing the role of the AARC concept with regard to survival.

In addition to AARC as a predictor of survival, we focused on two areas of psychosocial resources that have shown an association with survival in younger age groups and, therefore, may also be relevant for predicting survival in advanced old age. First, we addressed how very old individuals manage to maintain a self-view of a purposeful, valuable life, as seen from an individual and societal point of view. In a meta-analysis of ten prospective studies with more than 136,000 participants, Cohen et al. (2016) synthesized evidence for a robust link between purpose in life (e.g., being useful to others, life engagement, life meaning) and all-cause mortality. Similarly, the feeling of being needed was found to be the single most important aspect of positive life orientation to predict survival in a large Finnish study of community-dwelling individuals aged 75 years or older (Tilvis et al., 2012).

Second, another major psychological challenge is to what extent very old adults are able to exert control over their lives and keep track of current societal developments. This point addresses the core challenge whether and to what extent individuals in the Fourth Age can maintain feelings of agency and avoid feelings of being dependent on others. With respect to societal development, feeling distant and disconnected from major trends, such as globalization or communication technology, may result in possible alienation and perceived obsolescence. Adverse effects of alienation have been described in the context of suicide ideation in subpopulations with mood disorder (Moore, 1997). However, it is obvious to also expect more general negative consequences for late-life survival via reduced motivation for or limited perceived control over health-related behavior in very old age. Multiple studies have established an association between control beliefs and mortality hazard in older adult samples (Infurna et al., 2011, 2013; Wiest et al., 2013; Infurna and Okun, 2015; Duan-Porter et al., 2017; Hülür et al., 2017). For example, Infurna et al. (2011) noted that perceived control may be related to all-cause mortality through various mechanisms, including the absence of health promoting behaviors, ineffective emotion regulation, low social integration, and the absence of stress buffering effects. However, most studies considered a broad age range and used measures of perceived control administered as early as midlife to predict subsequent 8-, 11-, or 19-year mortality. Effects of control beliefs as a risk factor for mortality are likely to be different in a highly selected subpopulation of individuals that have already outlived most of their birth cohort peers. Because Infurna and Okun (2015) found that perceived control decreased with age and change in social participation, control beliefs may be changing substantially during the Fourth Age and more studies on changes in perceived control in very old individuals are needed. In addition, most studies have focused on the concept of internal control or mastery. We assumed that a multidimensional understanding of control that explicitly includes both feelings of mastery and dependency on powerful others or chance could be particularly helpful in very old age. Very old individuals may experience increased risk of chronic health conditions (Bercovitz et al., 2019), social losses, or a shifting potential for agency (Wahl et al., 2012), and therefore may become increasingly confronted with changes beyond their personal control.

The aims of this study were to examine the contribution of very old adults’ subjective VOA as predictors of survival time across a 3.5-year observation interval. Individuals’ subjective VOA, as operationalized in terms of AARC-Gains and AARC-Losses, were incorporated into a set of established socio-behavioral predictors of survival. We expected subjective VOA to show substantial associations with survival time. Moreover, we expected positive (AARC-Gains) and negative facets (AARC-Losses) of individuals’ subjective VOA to contribute independently to the prediction of survival in those in advanced old age, because these predictors have been shown to be differentially related to developmental outcomes in younger age groups. We expected a remaining increment of predictive power due to AARC even after controlling for other major survival-relevant psychological resources. Therefore, we assumed that attribution of perceived change to aging itself should evolve from a process of integrating knowledge of conditions more prevalent with age (i.e., associations with health status change) and experiences in handling such change (e.g., associations with perceived control).

## Materials and Methods

### Participants and Procedures

Data came from a representative panel study on quality of life (QoL) and well-being of very old adults conducted in Germany’s most populous state, North-Rhine Westphalia (Wagner et al., 2018). For the first wave of measurement in 2017/2018, a random community sample of persons aged 80 years and older was selected in a multi-stage sampling procedure, assuring adequate coverage of persons living both in private households and institutional settings. Persons in older age groups (i.e., 85 years and older) and men were oversampled to allow for precise estimation of population parameters also in these smaller subpopulations. A detailed discussion of the sampling design and efficiency as well as representativity of the weighted sample is available elsewhere (Hansen et al., 2021).

A total of 1,863 computer-assisted personal interviews were conducted at participants’ homes to assess a wide array of individual QoL resources (e.g., social and health) and subjective QoL outcomes (e.g., valuation of life). The study protocol also included objective testing such as a screening for mild cognitive impairment (MCI). Informed consent was given by all participants after written and verbal explanation of the study aims and procedures. Mean age of the realized sample at the time of the interview at wave 1 was 87.0 years (*SD* = 4.5 years; range: 80.1 to 102.9 years). A total of 211 interviews (11.3%) were conducted in nursing homes. The sample included 176 interviews with proxy informants (e.g., partner 48.3%, adult child 23.9%, other 27.8%) where target persons were willing to be included in the study but were not able to conduct the 90 min interview themselves due to severe mental or physical health constraints.

Based on consent, a total of 1,612 wave 1 participants were re-contacted in 2019/2020. Personal contacting during fieldwork revealed that 237 individuals had died since the first interview. Additional information on survival status and date of death was collected 1.5 years after wave 2 during regular panel maintenance work. By March 5, 2021 a total of 391 (24.2%) of respondents had died. The study was approved by the ethical board of the medical faculty at the University of Cologne (Protocol #: 17-169).

### Measures

#### Awareness of Age-Related Change

The 10-item short form of the Awareness of Age-Related Change scale (AARC-SF; Kaspar et al., 2019) was used as a brief measure of participants’ subjective VOA. The AARC-SF is multidimensional in capturing change across five behavioral domains: Health and physical functioning (PHYS), cognitive functioning (COG), interpersonal relations (INT), social-cognitive and social-emotional functioning (SC/SE), and lifestyle and engagement (LIFE). Half of the 10 items assess positive (gain-related) and half assess negative (loss-related) perceptions of age-related changes, respectively. The item stem is, “With my increasing age, I realize that …” and the response format ranges from 1 (*not at all*) to 5 (*very much*). A sample gain item (INT+ domain) is, “…I appreciate relationships and people much more.” A sample loss item (LIFE- domain) is, “…I have to limit my activities.” Kaspar et al. (2019) reported favorable psychometric properties and evidence for concurrent and discriminant validity. Composite reliability (see Revelle and Zinbarg, 2009) of the AARC-Gains and AARC-Losses scales in the current sample was acceptable given the built-in domain heterogeneity of the brief scales (MacDonald’s ω = 0.68 and 0.81, respectively).

#### Psychosocial Resources

Additional psychosocial resources predictive of survival in very old age were assessed in terms of appraisal of life (i.e., valuation of life, perceived obsolescence, feeling needed) and control (i.e., internal and external control foci).

The Valuation of Life Scale (VOL; Lawton et al., 2001) was used as a comprehensive measure of emotional and behavioral aspects of attachment to life in old age. The scale has 13 statements (e.g., “Life has meaning for me,” “I feel hopeful right now”) and a 3-point response scale 0 *(no)*, 1 *(neither/nor)*, 2 *(yes)* is suggested for use in very old respondents (Rott et al., 2001; Jopp et al., 2008, 2013). Gitlin et al. (2016) documented a two-factor structure of the VOL, which was replicated in the current sample. The two factors corresponded with an optimistic outlook in life (McDonald’s ω = 0.82) and personal engagement (McDonald’s ω = 0.87). Two items (i.e., “increasingly difficult to come to terms with today’s way of living”; “growing lack of fit between own values and those of society these days”) from the perceived obsolescence subscale of the Future Time Perspective Scale by Brandtstädter et al. (1997) and one item (i.e., “hard to stay oriented because society is changing so quickly these days”) from the anomia scale suggested by Gümüs et al. (2014) were used to measure respondents’ reflection of correspondence with values held by current society. Items were answered on a 4-point scale from 1 *(not at all)* to 4 *(very much)*. Scale consistency was moderate for this perceived obsolescence composite in the current sample (MacDonald’s ω = 0.69) and comparable to Cronbach’s alpha of 0.72 reported for the 5-item obsolescence subscale by Brandtstädter et al. (1997). In addition to individual values, subjective perceptions of appraisal by society were assessed using the single item “feel needed by society” with response options 1 *(not at all)* to 4 *(very much)*.

Perceived control was assessed using the Internal and External Control Beliefs scale (IE-4, Kovaleva et al., 2012), with two of the four items targeting internal control and two items targeting the external others and chance facets of external control beliefs suggested by Levenson (1972). Evidence of construct validity has been reported for this brief instrument by Kovaleva et al. (2012). Psychometric results for this dataset show both satisfactory consistency of the internal scale (MacDonald’s ω = 0.76) and uniqueness of the chance and powerful others indicators in the realm of external control beliefs, supporting a three factor interpretation.

#### Health and Cognitive Status

Adults’ self-reported performance on Basic Activities of Daily Living (ADL; Katz et al., 1963) and Instrumental Activities of Daily Living (IADL; Lawton and Brody, 1969) was used as a measure of everyday functioning. Specifically, we used five items to assess ADL (e.g., getting dressed and walking) and seven items to assess IADL (e.g., preparing meals and handling finances) with response options 0 *(not possible without help)*, 1 *(some help needed)*, 2 *(no help needed)*. Reliability of the ADL and IADL scales in the current sample was high (MacDonald’s ω = 0.92 and 0.93, respectively).

Further, the number of self-reported currently treated health conditions was used as an indicator of multimorbidity. Hence, this measure refers to a subset of medical conditions with high salience for the individual in everyday life. This index was modified from the Self-Administered Comorbidity Questionnaire (SCQ; Sangha et al., 2003) to include medical conditions particularly relevant in old age (Wiest et al., 2014). The 19 conditions included were: Heart disease (e.g., insufficiency), heart attack, hypertension, respiratory or lung disease, diabetes, gastrointestinal disease, kidney disease, liver disease, hemophilia (e.g., anemia), cancer, mental disease (e.g., phobia and depression), bone and joint disease (e.g., osteoporosis, arthrosis, and arthritis), back pain, urinary disorder, insomnia, hearing impairment, visual impairment, neurological disease (e.g., Parkinson’s and dementia), and stroke.

In terms of cognitive status, the DemTect was developed as a brief screening tool for MCI and early stages of dementia (Kalbe et al., 2004). The test has subtests assessing immediate/delayed word recall, digit span memory, number transformation, and verbal fluency. Favorable diagnostic properties in identifying beginning cognitive decline and MCI have been reported in comparison to alternative screening tools such as the MMSE (Kalbe et al., 2013) and age-specific scoring instructions for persons 80 years or older have been reported by Kessler et al. (2014). In the case of proxy interviews, cognitive status was reported with the Global Deterioration Scale (GDS; Reisberg et al., 1982) in seven stages from 1 *(no cognitive impairment)* to 7 *(most severe)*. Reisberg et al. (2011) aligned GDS stage 3 to correspond to a clinical presentation of MCI (Reisberg et al., 2011).

### Sample Selectivity

Characteristics of the very old population estimated based on (1) wave 1 participants, (2) those willing to be contacted again for future waves, and (3) respondents still alive approximately 3.5 years after wave 1 are shown in Table 1 to estimate potential selectivity of the analysis sample and risk factors to longevity.

**TABLE 1:** Characteristics of analysis samples with respect to initially reported socio-demographics and psycho-behavioral longevity indicators.

% [CI95] or M [CI95]	Wave 1		Post-study^a^
	Total (*N* = 1,863)	Gross panel sample (*n* = 1,612)	Survivors (*n* = 1,215)
**Gender (%)**			
– Men	36.3 [34.1–38.4]	36.3 [33.9–38.7]	36.0 [33.2–38.8]
– Women	63.7 [61.6–65.9]	63.7 [61.3–66.1]	64.0 [61.2–66.7]
**Age group (%)**			
– 80–84 years	54.3 [51.8–56.8]	55.6 [53.0–58.2]	59.9 [57.1–62.7]
– 85–89 years	30.7 [28.7–32.8]	30.6 [28.4–32.9]	29.8 [27.4–32.3]
– 90 years or older	15.0 [13.4–16.5]	13.8 [12.2–15.3]	10.3 [8.8–11.8]
**Living arrangement (%)**			
– Private	86.1 [83.6–88.6]	87.1 [84.7–89.5]	89.9 [87.6–92.3]
– Institution	13.9 [11.4–16.4]	12.9 [10.5–15.3]	10.1 [7.7–12.4]
**Views of Aging**			
AARC-Gains (1–5)	3.2 [3.1–3.2]	3.2 [3.1–3.3]	3.2 [3.2–3.3]
AARC-Losses (1–5)	2.8 [2.8–2.9]	2.8 [2.7–2.9]	2.7 [2.6–2.8]
**Health status**			
Treated health conditions (0–19)	3.5 [3.3–3.7]	3.5 [3.3–3.7]	3.4 [3.2–3.6]
ADL independence (0–2)	1.6 [1.6–1.7]	1.7 [1.7–1.7]	1.8 [1.7–1.8]
IADL independence (0–2)	1.4 [1.4 –1.4]	1.5 [1.4–1.5]	1.5 [1.5–1.6]
**Cognitive status^*b*^ (%)**			
– Age adequate function	68.1 [64.3–72.0]	70.6 [66.9–74.4]	74.3 [70.6–78.0]
– MCI	15.5 [13.3–17.7]	15.6 [13.2–17.9]	15.3 [12.8–17.8]
– Beginning AD	16.3 [13.4–19.2]	13.8 [11.2–16.4]	10.4 [8.2–12.6]
**Appraisal of life**			
VOL–Engagement (0–2)	1.4 [1.4 –1.5]	1.5 [1.4–1.5]	1.5 [1.5–1.6]
VOL–Optimism (0–2)	1.6 [1.6–1.6]	1.6 [1.6–1.6]	1.6 [1.6–1.6]
Perceived obsolescence (1–4)	2.5 [2.4–2.6]	2.5 [2.4–2.6]	2.5 [2.4–2.5]
Feeling needed by society (1–4)	2.2 [2.1–2.3]	2.2 [2.1–2.3]	2.2 [2.2–2.3]
**Perceived control**			
Internal (1–4)	3.3 [3.2–3.3]	3.3 [3.3–3.4]	3.4 [3.3–3.4]
Powerful others (1–4)	1.7 [1.6–1.7]	1.6 [1.5–1.7]	1.6 [1.5–1.6]
Chance (1–4)	2.2 [2.1–2.2]	2.2 [2.1–2.3]	2.2 [2.1–2.3]

*Weighted data. CI95, 95% confidence interval. ^*a*^According to panel management from March 5, 2021. ^*b*^In self-report interviews, 343 participants (20.3%) at wave 1 declined to take the cognitive screening test.*

Compared to the full wave 1 sample, the subsample of respondents willing to be contacted again for a second interview allowed for unbiased population estimates with respect to gender, multimorbidity, appraisal of life, perceived control, as well as AARC-Gains and AARC-Losses, as judged from almost completely overlapping 95% confidence intervals of parameter estimates in the initial and panel subsample. In addition, estimates for age composition (i.e., younger respondents), ADL/IADL independence, cognitive function and living arrangement (i.e., private) indicated no significant selectivity of those willing to be contacted again.

### Availability of Information on Survival Time

Information on survival status and survival time was collected across the study interval from different sources. Fieldwork metadata from contacting via mail and subsequent home visits or hotline responses from relatives included documentation of dropout due to death of respondents at wave 2, sometimes accompanied by more specific information (e.g., date). Additional postal registry data on survival status and date-of-death was obtained in March 2021 during panel maintenance. Of all 237 participants who had died since wave 1 according to information collected during wave 2 face-to-face fieldwork (i.e. home visits), valid death dates could be obtained for 141 individuals. Selectivity analyses found only very limited evidence of selective availability of death dates in this subsample of wave 2 non-respondents and potential bias in the subgroup with observed survival time (Table 2). More specifically, death dates were harder to obtain for individuals with lower ADL independence (OR = 5.4, 95% CI [1.1–26.6]) and stronger feelings of obsolescence (OR = 2.0, 95% CI [1.1–3.6]). However, the global Wald test for the multivariate prediction of data availability was non-significant (χ^2^ = 20.7, *df* = 18, *p* = 0.29).

**TABLE 2:** Selective availability of date-of-death in wave 2 non-respondents.

% [CI95] or M [CI95]	Wave 2	
	Deceased (*n* = 237)	Date-of-death (*n* = 141)
**Gender (%)**		
– Men	39.9 [32.5–47.3]	41.4 [31.0–51.7]
– Women	60.1 [52.7–67.5]	58.6 [48.3–69.0]
**Age group (%)**		
– 80–84 years	36.1 [27.6–44.6]	40.5 [29.9–51.1]
– 85–89 years	33.2 [25.9–40.5]	31.9 [22.2–41.7]
– 90 years or older	30.7 [24.4–36.9]	27.5 [19.5–35.6]
**Living arrangement (%)**		
– Private	67.4 [59.0–75.8]	71.0 [60.5–81.5]
– Institution	32.6 [24.2–40.9]	29.0 [18.5–39.5]
Views of aging		
AARC-gains (1–5)	3.0 [2.8–3.2]	3.1 [2.9–3.3]
AARC-losses (1–5)	3.2 [3.0–3.4]	3.2 [3.0–3.4]
**Health status**		
Treated health conditions (0–19)	3.8 [3.4–4.2]	3.8 [3.3–4.3]
ADL independence (0–2)	1.3 [1.2–1.4]	1.3 [1.2–1.4]
IADL independence (0–2)	0.9 [0.8–1.0]	1.0 [1.2–1.5]
**Cognitive status^*a*^ (%)**		
– Age adequate function	44.2 [34.6–53.7]	45.9 [33.8–58.0]
– MCI	17.1 [10.5–23.7]	16.4 [7.4–25.5]
– Beginning AD	38.7 [28.8–48.6]	37.6 [26.0–49.3]
**Appraisal of life**		
VOL–engagement (0–2)	1.3 [1.1–1.5]	1.3 [1.1–1.5]
VOL–optimism (0–2)	1.5 [1.4–1.6]	1.5 [1.4–1.6]
Perceived obsolescence (1–4)	2.6 [2.5–2.8]	2.6 [2.4–2.7]
Feeling needed by society (1–4)	2.1 [1.9–2.3]	2.2 [2.0–2.7]
**Perceived control**		
Internal (1–4)	3.0 [2.8–3.2]	3.1 [2.9–3.3]
Powerful others (1–4)	2.0 [1.8–2.2]	1.8 [1.6–2.1]
Chance (1–4)	2.3 [2.1–2.4]	2.2 [2.0–2.5]

*Weighted data, CI95, 95% confidence interval. ^*a*^In self-report interviews, 343 participants (20.3%) at wave 1 declined to take the cognitive screening test.*

**FIGURE 1:**
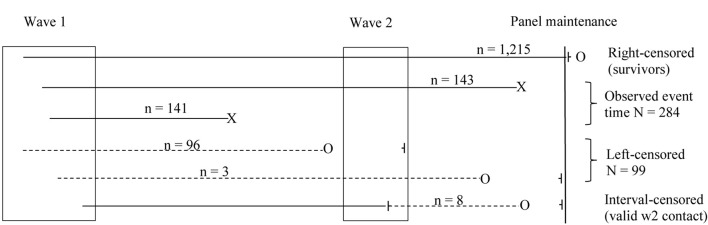
Study design and censoring scheme for survival time analysis. Observed survival times are indicated by X. Censored information on survival time is indicated by o ⊣.

Inability to retrieve the exact date-of-death for some respondents known to have died before wave 2 or by March 5, 2021, respectively, and the fact that most participants were still alive 3.5 years after initial assessment led to a complex scheme of censoring with respect to survival time in this study (Figure 1). A total of 1,215 individuals were still alive at the date of panel maintenance (March 5, 2021). Hence, only a lower bound of survival time for these individuals was observed and data were treated as censored from the right. Left-censoring (i.e., an upper-bound estimate of survival time) occurred where individuals were known to have died before wave 2 or panel maintenance, but no date of death could be retrieved (*n* = 96 and *n* = 3, respectively). Interval-censoring occurred for 8 cases where individuals were known to be alive at wave 2 (i.e., from documented contact) and deceased by March 5, 2021 (i.e., from registry data), but no death date could be obtained.

### Plan of Analysis

In a first step, we describe levels of socio-behavioral factors of longevity in a subsample of wave 1 respondents known to have survived at least 3.5 years from the initial interview.

In a second step, we used accelerated survival time analysis (also referred to as accelerated failure time AFT; Pike, 1966) to identify risks to and protective factors for longevity across a study interval of up to 3.5 years after the first interview. Although originally developed for time-to-event analyses in economics and engineering, AFT has been proven particularly useful for predicting survival time (for review, see Saikia and Barman, 2017). AFT is a fully parametric model that uses the log of survival time as dependent variable and specific distributional assumptions about the error terms:


$$\log\left(T_{i}\right)=\beta_{0}+\beta_{1}\,z_{i\,1}+\ldots +\beta_{p}\,z_{i\,p}+\sigma \,\varepsilon_{i}$$

Compared to the widely used semi-parametric Cox proportional hazard (PH) model, the parametric AFT model offers more efficient estimation (i.e., narrower confidence intervals) when distributional assumptions are met. Whereas, semi- or non-parametric models offer a limited range of possible censoring schemes, AFT can handle interval- and left-censored time-to-event data in addition to the more common right-censored scenario (Kalbfleisch and Prentice, 2002). Hence, it uses all available information on survival time from all wave 1 respondents that gave consent to be contacted again for wave 2 (see Figure 1) in estimating risk factors for mortality in this study of the very old. Moreover, AFT offers a straightforward interpretation of parameters because predictors are directly and multiplicatively linked to survival time. Taking exp (β_p_) gives the event-time-ratio (ETR) that represents the factor by which time-to-death itself is accelerated (i.e., shortened) or slowed down (i.e., elongated) in a “treatment” group compared to a reference group. Hence, an ETR of 1.5 describes a protective factor that increases survival time by 50 % compared to that in the reference group, whereas, an ETR of 0.75 describes a risk factor that reduces survival time to 75 % of that in the reference group. Inspection of the cumulative distribution function of time-to-death for different distributional assumptions in an intercept-only model indicated that both the Weibull and log-normal function may be used to fit the data, but that the log-normal function was better suited to model shorter survival time (see Supplementary Figure 1). The inverted U-shape of the log-normal hazard appears to be particularly appropriate for the present data, because immediately after the wave 1 interview, the hazard for mortality was likely to be low and then increased with time. The fact that the log-normal hazard eventually began to decline accommodates a potential selectivity of “survivors” of very old individuals far beyond their cohort’s life expectancy, and the fact that the interim wave 2 fieldwork may have been more effective in identifying deceased individuals than the registry-based panel maintenance (see Figure 1).

All survival analyses controlled for socio-demographic background characteristics, such as age, gender, or living in an institution, and used participants’ subjective VOA, life appraisals, control beliefs as well as cognitive and health resources as predictors. Predictors were introduced block-wise in a predetermined order to estimate the unique relevance of VOA among the behavioral indicators of survival and the robustness of parameter estimates.

All analyses also include available proxy information. As to be expected, our study was faced with a classic trade-off: On the one hand, it may be seen critical to involve external individuals also when it comes to self-referential information such as the AARC-SF. However, we always involved proxy persons that were very familiar with the target individual. On the other hand, doing so can be seen as a needed strategy to counteract a likely under-representation of a seemingly most vulnerable part of the very old population. Besides such a meta-methodological argument, we were able to establish measurement equivalence of the AARC-Gains and AARC-Losses scales across self- and proxy-report using multi-group structural equation modeling with equality constraints (see Supplementary Table 1).

All analyses used calibration weights to correct for the disproportional sampling design and survey nonresponse at wave 1 to allow for unbiased population estimates (Valliant et al., 2013). All analyses were performed using SAS 9.4 (SAS Institute, Cary, NC) software.

## Results

### Socio-Behavioral Characteristics of Survivors in Very Old Age

The subgroup of study participants alive 3.5 years later (see Table 1) initially reported significantly more AARC-Gains (*M* = 3.2, 95% CI [3.2–3.3]) than AARC-Losses (*M* = 2.7, 95% CI [2.6–2.8]). High average initial levels were found for the VOL subscales, indicating considerable engagement with life and optimism in very old age (1.5 and 1.6 points on the 0 to 2 points response scale, respectively). Nevertheless, the average level of feelings of obsolescence and anomia observed in this sample was close to scale midpoint (*M* = 2.5, 95% CI [2.4–2.5]), indicating a fair amount of perceived discrepancy of values and lifestyle in today’s very old adults compared to current society. In a related vein, most respondents showed only moderate levels of feeling needed by society (*M* = 2.2, 95% CI [2.2–2.3]). With respect to health and functioning, the survivor subgroup reported, on average, 3.4 treated health conditions (95% CI [3.2–3.6]) as an indication of multimorbidity. Need for assistance with basic ADL was lower (*M* = 1.8, 95% CI [1.7–1.8]) than need for assistance with more complex IADL (*M* = 1.5, 95% CI [1.5–1.6]). Almost three out of four respondents in the survivor group showed age-adequate cognitive function in the screening, whereas, only one out of 10 respondents in this group were screened for probable beginning AD at the initial interview. Hence, the survivor subsample had better cognitive functioning than the average population 80+ with respect to the estimated prevalence of cognitive impairment (Doblhammer et al., 2012) and higher level of independence in ADL/IADL (see section “Sample Selectivity”).

### Awareness of Age-Related Change and Survival Time

For the 284 individuals with available death dates, observed survival time after initial interview was 612.5 days (range 2 to 1,259 days). Because fieldwork for initial interviews span 196 days, survival time for those 1,215 participants still alive on March 5, 2021 was 1,201.5 days on average, with a range from 1,105 to 1,301 days.

Estimated event time ratios (ETR) from the AFT model are given in Table 3 for different sets of socio-behavioral predictors of survival. These predictors were included in a pre-determined order (i.e., background variables, VOA, appraisal of life, perceived control, and health resources) to estimate the unique contribution of each set of predictors and examine change in predictive value when competing predictors were added to the analysis. AARC-Gains and AARC-Losses were added right after control variables due to the special emphasis on the role of VOA for late-life survival.

**TABLE 3:** Results of accelerated failure time model: Predicting survival time using VOA as socio-behavioral indicator of longevity.

Predictor variables	Accelerated failure time (AFT) model				
	Model 1 (*n* = 1,606)	Model 2 (*n* = 1,592)	Model 3 (*n* = 1,484)	Model 4 (*n* = 1,360)	Model 5 (*n* = 1,154)
	ETR [CI95]	ETR [CI95]	ETR [CI95]	ETR [CI95]	ETR [CI95]
**Socio-demographics**					
Gender (ref.: Women)	**0.74 [0.58–0.94]**	**0.73 [0.58–0.92]**	**0.75 [0.59–0.96]**	**0.72 [0.55–0.94]**	**0.73 [0.55–0.98]**
**Age group (ref.: 90 years or older)**					
– 80–84 years	**3.01 [2.16–4.20]**	**2.64 [1.90–3.68]**	**2.61 [1.85–3.69]**	**2.77 [1.88–4.07]**	**2.32 [1.52–3.55]**
– 85–89 years	**2.01 [1.44–2.81]**	**1.89 [1.36–2.63]**	**1.88 [1.33–2.67]**	**1.80 [1.23–2.64]**	**1.65 [1.09–2.49]**
Institutionalization (ref.: Private)	**0.42 [0.31–0.58]**	**0.58 [0.42–0.81]**	**0.56 [0.40–0.80]**	**0.64 [0.43–0.96]**	1.02 [0.64–1.64]
**Views of aging**					
AARC-Gains (1–5)		**1.27 [1.11–1.46]**	**1.30 [1.11–1.51]**	**1.27 [1.08–1.50]**	1.13 [0.94–1.35]
AARC-Losses (1–5)		**0.72 [0.63–0.81]**	**0.67 [0.57–0.78]**	**0.68 [0.57–0.80]**	**0.81 [0.66–0.98]**
**Appraisal of life**					
Valuation of life–engagement (0–2)			0.88 [0.69–1.14]	**0.70 [0.51–0.95]**	0.71 [0.50–1.01]
Valuation of life–optimism (0–2)			1.02 [0.71–1.46]	1.07 [0.71–1.61]	0.91 [0.58–1.44]
Perceived obsolescence (1–4)			1.00 [0.86–1.15]	1.05 [0.89–1.23]	0.97 [0.81–1.15]
Feeling needed by society (1–4)			0.93 [0.82–1.04]	0.90 [0.79–1.03]	0.88 [0.77–1.01]
**Perceived control**					
Internal (1–4)				1.11 [0.88–1.39]	1.03 [0.80–1.34]
Powerful others (1–4)				0.85 [0.72–1.01]	0.90 [0.74–1.09]
Chance (1–4)				1.12 [0.98–1.28]	**1.18 [1.02–1.37]**
**Health resources**					
Treated health conditions (0–19)					0.99 [0.93–1.06]
ADL independence (0–2)					**1.75 [1.08–2.82]**
IADL independence (0–2)					1.33 [0.86–2.07]
**Cognitive status (ref.: Beginning AD)**					
– Age adequate function					1.57 [0.98–2.53]
– MCI					1.19 [0.71–2.01]
Scale	**1.65 [1.49– 1.83]**	**1.61 [1.45**–**1.79]**	**1.61 [1.44**–**1.79]**	**1.65 [1.47**–**1.86]**	**1.60 [1.40**–**1.82]**
AIC/BIC	2073.70/2106.07	2011.78/2054.88	1847.08/1937.97	1670.42/1749.03	1346.99/1448.83

*Weighted data. ETR, event time ratio; CI95, 95% confidence interval. Parameter estimates significant at the *p* < 0.05 level are shown in bold font.*

Results showed a rather consistent pattern of associations of socio-demographic background variables with 3.5-year survival in very old age across all models. Specifically, an estimated ETR of 0.72 to 0.75 for men indicated that survival time was 25 to 28 percent shorter in very old men compared to women. Younger age was also consistently and positively associated with survival time. Expected survival time was 65% longer in the 85–89 age group than that for the oldest-old (90+), and 132% longer in the youngest participants (80–84 years). Acceleration of time-to-death was observed for respondents in institutional settings (i.e., 36% to 58% shorter survival time) compared to community-residing participants in all models except model 5 that also included physical and cognitive health status as predictors.

Awareness of age-related gains (AARC-Gains) and AARC-Losses were independent and significant predictors of survival time over and above socio-demographic background characteristics (Model 2). Reporting more AARC-Gains at wave 1 was associated with longer survival time, whereas, reporting more AARC-Losses was associated with a reduction in survival time of approximately comparable magnitude. Adding psychosocial resources in the following models, effects of VOA on estimated survival time stayed virtually the same when different aspects of appraisal of own life (Model 3) and different facets of perceived control over life (Model 4) were added to the list of predictors. Notably, AARC-Losses remained a significant independent predictor of survival in the oldest-old even when multimorbidity, ADL/IADL independence and cognitive status were controlled for (Model 5). However, AARC-Gains no longer was a significant independent predictor in the full set of socio-behavioral factors of survival. Of note, parameter estimates were virtually unaltered in subsequent sensitivity analyses that excluded proxy reports (see Supplementary Table 2).

Results for the set of predictors tapping appraisals of one’s own life were both less consistent across models with different competing predictors and showed smaller associations with survival time. The ERT for the “engagement with life” subscale of the VOL scale was consistently estimated below 1.0 (0.88, 0.70, and 0.71), indicating shorter survival time after initial interview in those reporting feelings of self-efficacy in overcoming problems and difficult situations. However, a significant effect could only be observed after controlling for perceived control (Model 4), suggesting some degree of overlap between engagement with and perceived responsibility for one’s life. Results also suggested a potential marginal association between feeling needed by society and reduced survival time with ERTs of 0.93, 0.90 and 0.88, respectively. These effects, however, failed to reach statistical significance in all models.

Results with respect to different facets of perceived control in very old age were mixed. Neither internal nor external control beliefs contributed significantly to the prediction of late-life survival unless effects of health resources were controlled for in Model 5. Specifically, once health resources were taken into account, respondents who reported higher dependency of personal life outcomes on chance or luck (rather than on own action or those of powerful others) showed significantly longer survival times (ERT: 1.18) after the initial interview.

Of the proposed health and functioning predictors of mortality, only basic ADL independence was found to be significantly associated with survival time in this sample. A 1-point increase in ADL performance (e.g., 0 = *Not possible without help* to 1 = *Some assistance needed*) was associated with a 75% increase in survival time (ERT: 1.75). Whereas, estimates for IADL and levels of cognitive functioning also pointed in the expected direction, benefits of age-adequate cognitive functioning for survival time did not meet the.05 level of statistical significance. It is noteworthy that considering health status differences at the initial interview in Model 5 significantly attenuated some of the effects reported for less comprehensive models. Not surprisingly, the effect of living in an institution on survival time diminished when taking health and functional status into account. Similarly, the protective effect of positive VOA and the adverse effect of engagement with life on survival time became non-significant when health differences were controlled for.

## Discussion

The aim of this study was to test the predictive relevance of very old adults’ subjective VOA vis-à-vis other socio-behavioral indicators from the domains of appraisal of life, perceived control, and health.

Adding to the extant previous literature of increased mortality risks in those with more negative VOA, this study found that perceived AARC-Gains and AARC-Losses predicted survival time across a 3.5-year observational interval in a large representative sample of respondents aged 80 years or older. Importantly, AARC-Gains and AARC-Losses were found to be significant and independent predictors of late-life survival. These effects remained significant across a wide range of competing socio-behavioral indicators, including control beliefs, valuation of own life, perceived obsolescence, and perceived appreciation by society for which substantial effects on mortality had been reported in previous studies (Infurna et al., 2011, 2013; Tilvis et al., 2012; Wiest et al., 2013; Infurna and Okun, 2015; Hülür et al., 2017; Alimujiang et al., 2019). Moreover, the predictive value of AARC-Losses for shorter survival time remained unaltered when ADL/IADL independence, multimorbidity and cognitive functioning were taken into account. This finding is in line with that of Kotter-Grühn et al. (2009), who found effects of aging satisfaction and subjective age on mortality hazard to be robust when controlling for age, gender, socioeconomic status, diagnosis of dementia, or number of illnesses. However, this study is the first to show that different dimensions of very old adults’ subjective VOA were differently related to survival. Moreover, findings from this study also showed that it may be premature to assume that very old adults’ subjective VOA are exclusively focused on age-related losses.

In contrast, the predictive value of engagement with life and living in an institution appeared to be less independent from health status than VOA. This finding lends support to the idea that the higher likelihood of impairment in the Fourth Age may indeed level out mortality risks apparent in younger age groups. Thus, and the “age-as-leveler” hypothesis discussed by Goldman et al. (2017) for social factors seems to also apply to psychological predictors of survival. Results also suggested that some of the protective effect of perceived AARC-Gains may in fact be attributable to differences in health status or may be restricted to individuals who retain good health into very old age. However, the latter interpretation was not supported by the data, judged from a non-significant estimate of an additional ADL × AARC-Gains term and worse fit of this extended model. Therefore, we conclude that perceived age-related gains may have limited potential to buffer health-related mortality risk in this specific age group. Rather, AARC-Gains appear to be dependent on retained health and function in very old age that allow for continuity of activities and social engagement (Kaspar et al., 2021). Hence, results from the multidimensional measure of VOA suggest that interventions may be tailored to promote late-life survival via different channels and under different health conditions. For example, awareness of age-related gains may be promoted efficiently as part of activity-based intervention programs (Beyer et al., 2019), whereas, dysfunctional awareness of age-related losses may be addressed using elements of cognitive behavioral therapy (Wolff et al., 2014). At the same time, results indicate that AARC-Gains have smaller leverage than AARC-Losses in supporting late-life survival. Moreover, their benefits seem to depend more strongly on functional health status. This suggests that interventions to increase awareness of age-related gains may be particularly useful in very old individuals with low or moderate risks to survival. Another practical implication of our findings is that subjective aging indicators such as AARC may also become part of classic comprehensive geriatric assessment (Stuck et al., 1993) in that they signal heightened health risks (see Kaspar et al., 2021) and at least indirectly also all-cause mortality risks.

The result that higher engagement with life from the valuation of life scale and to some extent also the feeling of being needed by society were negatively associated with survival time may appear counterintuitive because positive life orientation, including purpose in life and feeling needed, has been found to be associated with lower mortality risk in other studies (Tilvis et al., 2012; Windsor et al., 2015; Alimujiang et al., 2019). However, in very old age both indicators may signal an intensifying struggle with the increasingly adverse conditions of late life rather than representing a set of drivers for agency or motives for engagement that very old individuals would still be able to fully live up to. This interpretation is in line with findings by Windsor et al. (2015) showing that associations between purpose in life and survival became weaker over time. Hence, the findings reported here corroborate their conclusion that a higher sense of purpose may not buffer against more pervasive losses in health that become more common in very late life.

With respect to perceived control, we found no significant predictive effect of internal control on survival time in this sample of very old individuals. Whereas this finding is different from those of previous studies in younger age groups, Hülür et al. (2017), based on a sample from the Socioeconomic Panel (SOEP) study (mean age 44.4 years at baseline, range 18 to 98 years), reported a substantial decrease in the predictive power of perceived control (i.e., mastery) for mortality in individuals who exhibited accelerated decline in life satisfaction. Such an accelerated decline in life satisfaction tends to be a characteristic of the terminal phase in life (Gerstorf et al., 2010). For example, Hülür et al. (2017) concluded that when life circumstances are especially difficult, perceived control may not bring an additional advantage, because chances for goal attainment may be rather low (Heckhausen et al., 2013). Moreover, results of the current study suggest a possible protective effect of fatalism (i.e., external control/chance) on survival–at least in concert with an increased risk of dying in those with more prominent health impairments. Thus, we speculate that acknowledging the natural course of life and accepting limited opportunities for agency in very late life may have been beneficial in this sample of very old individuals.

Our finding that basic ADL, but not instrumental ADL was related to late-life survival is consistent with that of Goldman et al. (2017) that the unique predictive value of IADL for survival is greatly decreased in very old age, whereas that of basic ADL is retained in very old age compared to younger age groups. It is also consistent with Lawton and Brody’s (1969) view that ADLs and IADLs can be arranged in a hierarchy of functioning. If the ability to perform basic ADLs becomes compromised in a very old individual, this should be viewed as a signal of a stronger mortality hazard and at the same time imply significant limitations in more complex tasks of everyday activity.

Taken together, risk factors to late-life survival reported here for a representative sample of individuals beyond the age of 80 were in part different from those reported in previous studies using more age-diverse samples. In our view, these differences emphasize the bounded nature of socio-behavioral adaptation because of increasingly complex health and social conditions in very old and oldest age. Hence, diminishing health and social resources that characterize Fourth Age may potentially render ineffective or even counterproductive some socio-behavioral determinants of longevity known to be effective in younger groups, such as feelings of being needed or in control.

### Limitations and Outlook

Several shortcomings of this study need to be acknowledged. Although results are based on a representative sample of very old individuals, most study participants were still alive at the end of the 3.5-year observation interval. Hence, longer observational intervals may be needed even in studies of the oldest-old to capture end-of-life dynamics. Second, although data from fieldwork of an intermediate second wave was used to define survival status and survival time, substance-matter results of the second wave of realized interviews have not been integrated in this study. In this respect, future work should examine the benefits of considering change in perceived age-related gains and losses, along with change in health status and other socio-behavioral determinants, as predictors of survival time.

Overall, the findings of the present study help to shed some light on the delicate balance between the extraordinary health-related, psychological and social challenges of the Fourth Age and the availability of personal and contextual resources to cope with these challenges. We believe that assessing and understanding very old individuals’ subjective VOA, which at that time have reflected ongoing development and identity over long periods of their lifetime, may help to gain a better understanding of expectations of and socio-behavioral responses to late-life survival. Moreover, study results underscore the need for intervention studies to sensitize individuals to awareness of age-related changes to promote timely and sustainable changes in health behavior or adaptation that could foster longevity. We have shown elsewhere (Kaspar et al., 2021) that AARC is both a predictor of future health status and a consequence of health changes over time; thus, indicating a reciprocal association over time. Adding to intervention studies that seek to reduce age stereotype threat (Levy et al., 2014), the findings reported here suggest a potential benefit of promoting awareness of age-related gains for late-life survival.

## Data Availability Statement

The datasets presented in this study can be found in online repositories. The names of the repository/repositories and accession number(s) can be found below: GESIS Datenarchiv, Köln. ZA7558 Datenfile Version 1.0.0, https://doi.org/10.4232/1.13527.

## Ethics Statement

The studies involving human participants were reviewed and approved by the ethical board of the medical faculty at the University of Cologne (Protocol #: 17-169). Written informed consent for participation was not required for this study in accordance with the national legislation and the institutional requirements.

## Author Contributions

RK initiated the study, collected the data, conducted the analyses, and wrote the manuscript. H-WW and MD wrote the manuscript. All authors contributed to the article and approved the submitted version.

## Conflict of Interest

The authors declare that the research was conducted in the absence of any commercial or financial relationships that could be construed as a potential conflict of interest.

## Publisher’s Note

All claims expressed in this article are solely those of the authors and do not necessarily represent those of their affiliated organizations, or those of the publisher, the editors and the reviewers. Any product that may be evaluated in this article, or claim that may be made by its manufacturer, is not guaranteed or endorsed by the publisher.

## Supplementary Material

Supplementary Figure 1 Visual inspection of fit for log normal and Weibull distribution of error terms.

Supplementary Table 1 Measurement equivalence of AARC-Gains and AARC-Losses across self- and proxy report.

Supplementary Table 2 Sensitivity analysis: Predicting survival time using VOA as socio-behavioral indicator of longevity in self-report interviews only.
