# Supplementary material for: Awareness of Age-Related Change as a Behavioral Determinant of Survival Time in Very Old Age
Source: Front Psychol. 2021 Sep 28;12:727560. doi: 10.3389/fpsyg.2021.727560 (PMC8505719; doi:10.3389/fpsyg.2021.727560)
Supplement: Supplementary Figure 1 — Visual inspection of fit for log normal and Weibull distribution of error terms. [file Data_Sheet_1.docx]

Supplementary Material

**1 Measurement Invariance of the AARC-10 SF across Self- and Proxy Report**

Analysis of survival time included available proxy information in case the targeted individual was unable to respond to avoid under-representation of this most vulnerable part of the population in the current study. Towards this goal, we employed multi-group structural equation modeling with equality constraints to test for measurement equivalence between self- and proxy-report. Results of the test for measurement equivalence of the AARC scales are shown in Supplementary Table 1. The pattern of factor loadings of indicators was the same across self- and proxy report for age-related gains. For age-related losses, the pattern of loadings was also equivalent for all but one indicator. More specifically, in communication-impaired individuals represented by proxy interviews, the cognitive health domain (COG- “mental capacity is declining”) was more strongly related to AARC-Losses than it was in individuals able to conduct the interview themselves. Whereas the “meaning” of the gain and loss factors were very similar across proxy and self-report, more prominent differences were found between subgroups with respect to the use of response categories (i.e. response shift). For age-related losses, the intercept of item COG- (“mental capacity is declining”) was estimated lower in proxy compared to self-report interviews, i.e. these experiences were less often/easily reported by proxies irrespective of the estimated total level of perceived age-related loss. An opposite deviation was found in proxy interviews for items INT- (“more dependent on help of others”) and SCSE- (“harder to motivate”), which were more often/easily reported than it was at the same level of age-related loss in self-report interviews. For age-related gains, intercepts for items PHYS+ (“pay more attention to health”) and LIFE+ (“more freedom to live days the way wanted”) were lower in the proxy group, thus these experiences were reported less often/easily at the same level of age-related gains than in self-report. An opposite deviation was found for item INT+ (“appreciate relationships and people much more”) that received higher ratings in proxy interviews than in self-report, irrespective of the respective level of age-related gains. The pattern of differences found between AARC ratings for people able to conduct the interview themselves (self-report) and those unable to do so (proxy report) were plausible in that it suggested that proxy informants referred more strongly to observable characteristics such as the compromised health status of the persons concerned (e.g. attention to health, declining mental capacity) or characteristics of their interaction with them (e.g. motivation, help by others, appreciation).

With respect to the comparability of scores across groups, we make two observations. First, because metric equivalence (i.e. comparable factor loads) could be established for all AARC-Gains items and all but one AARC-Loss items, results of using these subscales in covariance-based analysis such as AFT used in this study will be unbiased. Second, whereas some items were overrated, others were underrated in the proxy group compared to the self-report group. Thus, some of these effects will cancel each other out in aggregate scores. Nevertheless, our results indicated that observed scale scores would overall be biased to be lower with respect to gains and higher with respect to losses in proxy report.

**Supplementary Table 1.** Measurement equivalence of AARC-Gains and AARC-Losses across self- and proxy report.

| Estimate (S.E.) | | Loading | | Intercept | |
| --- | --- | --- | --- | --- | --- |
| Domain^1^ | With my increasing age, I realize that … | Self-report | Proxy report | Self-report | Proxy report |
| INT+ | …I appreciate relationships and people much more. | 0.82 (0.06) | same | 0.22 (0.18) | 0.72 (0.17) |
| PHYS+ | …I pay more attention to my health. | 0.77 (0.06) | same | 0.99 (0.21) | 0.54 (0.17) |
| COG+ | …I have more experience and knowledge to evaluate things and people. | 1.31 (0.07) | same | -1.07 (0.21) | same |
| SCSE+ | …I have a better sense of what is important to me. | 1.37 (0.05) | same | -1.07 (0.17) | same |
| LIFE+ | …I have more freedom to live my days the way I want. | 0.73 (0.06) | same | 0.93 (0.18) | 0.47 (0.19) |
| COG- | …my mental capacity is declining. | 0.57 (0.04) | 1.09 (0.15) | 0.68 (0.11) | -0.15 (0.51) |
| LIFE- | …I have to limit my activities. | 1.22 (0.03) | same | 0.01 (0.08) | same |
| PHYS- | …I have less energy. | 1.17 (0.03) | same | -0.03 (0.07) | same |
| INT- | …I feel more dependent on the help of others. | 1.21 (0.03) | same | -0.72 (0.09) | 0.02 (0.15) |
| SCSE- | …I find it harder to motivate myself. | 0.83 (0.03) | same | 0.06 (0.09) | 0.31 (0.16) |

Note. ^1^AARC Domain Abbreviations: PHYS = Health and Physical Functioning; COG = Cognitive Functioning; INT = Interpersonal Relations; SCSE = Social-Cognitive and Social-Emotional Functioning; LIFE = Lifestyle and Engagement. ‘+’ positive domains; ‘-’ = negative domains.

**2 Choice of Distributional Assumption for Error Terms in Accelerated Failure Time Model**

Panel (A) Panel (B)


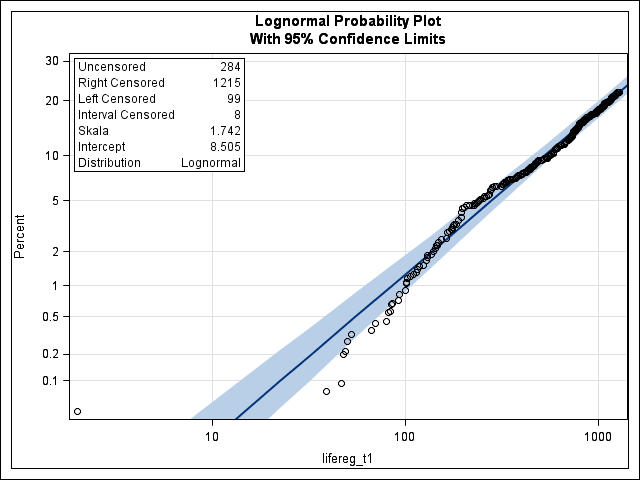

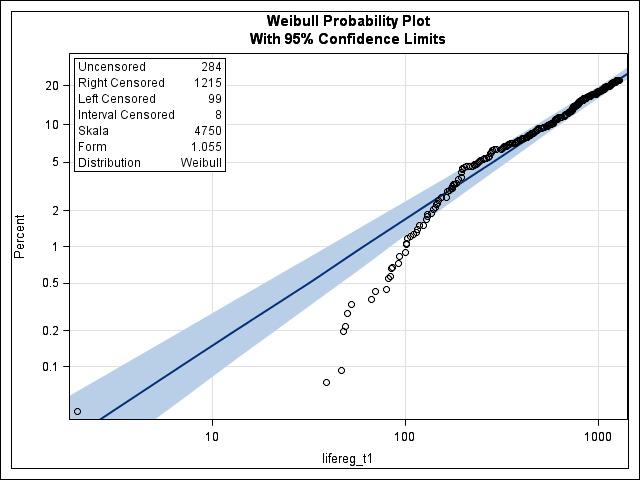


**Supplementary Figure 1.** Visual inspection of fit for log normal (A) and Weibull (B) distribution of error terms. Assuming a lognormal distribution of error terms in the fully parametric Accelerated Failure Time (ATF) model is more appropriate in representing the survival time data than assuming a Weibull distribution. Lifereg_t1 = survival time since wave 1 interview in days.

**3 Sensitivity Analysis**

**Supplementary Table 2.** Sensitivity analysis: Predicting survival time using VOA as socio-behavioral indicator of longevity in self-report interviews only.

| Predictor Variables | Accelerated Failure Time (AFT) model | | | | |
| --- | --- | --- | --- | --- | --- |
|  | Model 1 (n=1,514) | Model 2 (n=1,509) | Model 3 (n=1,418) | Model 4 (n=1,311) | Model 5 (n=1,106) |
|  | ETR [CI95] | ETR [CI95] | ETR [CI95] | ETR [CI95] | ETR [CI95] |
| **Socio-demographics** |  |  |  |  |  |
| Gender (ref.: Women) | **0.76 [0.60 - 0.98]** | **0.75 [0.59 - 0.96]** | 0.78 [0.60 - 1.01] | **0.75 [0.57 - 0.99]** | 0.77 [0.57 - 1.04] |
| Age group (ref.: 90 yrs or older) |  |  |  |  |  |
| - 80-84 yrs | **2.97 [2.08 - 4.23]** | **2.64 [1.86 - 3.75]** | **2.58 [1.79 - 3.73]** | **2.63 [1.76 - 3.93]** | **2.22 [1.42 - 3.47]** |
| - 85-89 yrs | **1.97 [1.38 - 2.81]** | **1.88 [1.32 - 2.67]** | **1.87 [1.29 - 2.72]** | **1.81 [1.21 - 2.70]** | **1.65 [1.06 - 2.58]** |
| Institutionalization (ref.: Private) | **0.50 [0.35 - 0.72]** | **0.63 [0.44 - 0.90]** | **0.60 [0.41 - 0.88]** | 0.69 [0.45 - 1.05] | 1.03 [0.62 - 1.71] |
| **Views of Aging** |  |  |  |  |  |
| AARC-Gains (1-5) |  | **1.21 [1.04 - 1.41]** | **1.25 [1.06 - 1.47]** | **1.22 [1.03 - 1.45]** | 1.10 [0.91 - 1.33] |
| AARC-Losses (1-5) |  | **0.74 [0.65 - 0.85]** | **0.68 [0.58 - 0.80]** | **0.69 [0.58 - 0.82]** | 0.81 [0.65 - 1.01] |
| **Appraisal of life** |  |  |  |  |  |
| Valuation of life – Engagement (0-2) |  |  | 0.77 [0.58 - 1.02] | **0.64 [0.46 - 0.88]** | **0.67 [0.46 - 0.97]** |
| Valuation of life – Optimism (0-2) |  |  | 1.06 [0.71 - 1.57] | 1.09 [0.71 - 1.67] | 0.91 [0.56 - 1.47] |
| Perceived obsolescence (1-4) |  |  | 1.04 [0.89 - 1.22] | 1.09 [0.92 - 1.29] | 0.99 [0.83 - 1.19] |
| Feeling needed by society (1-4) |  |  | 0.94 [0.83 - 1.07] | 0.92 [0.81 - 1.05] | 0.90 [0.78 - 1.04] |
| **Perceived control** |  |  |  |  |  |
| Internal (1-4) |  |  |  | 1.17 [0.92 - 1.49] | 1.13 [0.86 - 1.48] |
| Powerful others (1-4) |  |  |  | 0.90 [0.75 - 1.08] | 0.92 [0.75 - 1.13] |
| Chance (1-4) |  |  |  | 1.07 [0.93 - 1.22] | 1.13 [0.96 - 1.32] |
| **Health resources** |  |  |  |  |  |
| Treated health conditions (0-19) |  |  |  |  | 1.00 [0.93 - 1.06] |
| ADL independence (0-2) |  |  |  |  | **1.75 [1.03 - 2.98]** |
| IADL independence (0-2) |  |  |  |  | 1.29 [0.81 - 2.05] |
| Cognitive status (ref.: Beginning AD) |  |  |  |  |  |
| - Age adequate function |  |  |  |  | 1.43 [0.86 - 2.38] |
| - MCI |  |  |  |  | 1.06 [0.61 - 1.86] |
| Scale | **1.66 [1.49– 1.86]** | **1.63 [1.46 – 1.82]** | **1.64 [1.46 – 1.83]** | **1.66 [1.47 – 1.88]** | **1.62 [1.41 – 1.86]** |
| AIC/BIC | 1865.19/1897.24 | 1841.95/1884.66 | 1731.06/1794.46 | 1574.82/1652.97 | 1266.34/1367.49 |

Note. Weighted data. ETR = Event Time Ratio, CI95 = 95% Confidence Interval. Parameter estimates significant at the *p* < .05 level are shown in bold font.
